# Striking a balance between in-person care and the use of eHealth to support the older rural population with chronic pain

**DOI:** 10.3402/qhw.v10.27536

**Published:** 2015-09-02

**Authors:** Anne Roberts, Lorna Philip, Margaret Currie, Alasdair Mort

**Affiliations:** 1 Centre for Rural Health, University of Aberdeen, Aberdeen, UK; 2 Department of Geography and Environment, University of Aberdeen, Aberdeen, UK; 3 Social, Economic and Geographical Sciences Group, The James Hutton Institute, Aberdeen, UK

**Keywords:** eHealth, technology, health care, rural, older people, chronic pain, social interaction

## Abstract

New and existing information communication technologies (ICT) are playing an increasingly important role in the delivery of health and social care services. eHealth^1^ has the potential to supplement in-person home visits for older, rural adults with chronic pain. The Technology to support Older Adults' Personal and Social Interaction project—TOPS—examines interactions between older people and their health/social care providers and considers how eHealth could play a part in enhancing the life experiences of older people with chronic pain, who live in remote/rural areas. This paper reports findings from the TOPS study, drawing upon observations of health/social care home visits to chronic pain patients and interviews with patients and health/social care providers in rural Scotland. Patients and care professionals believe in-person care promotes the general well-being of older people with pain. However, our findings show that the potential recipients of eHealth are open to the use of such technologies and that although they cannot be expected to replace existing models of care, eHealth may provide opportunities to sustain and enhance these interactions.

There is considerable potential for eHealth to support ageing in place, offering innovative and potentially more cost-effective means of delivering a range of in-home health and social care services. Social isolation, older adults, chronic pain, health and social care, and new eHealth technologies provide the context for ongoing research funded as part of the Research Council's UK Digital Economy programme through the dot. rural research hub at the University of Aberdeen (see Mort & Philip, 2014, for further details). The Technology to support Older Adults' Personal and Social Interaction project (hereafter TOPS) is concerned with the social and personal interaction between older people and their health/social care providers and considers how eHealth could contribute appropriately to enhancing the life experiences of older people with chronic pain living in rural areas.

In this paper, we report findings from qualitative research involving older adults with chronic pain and their health/social care professionals who live and work in a remote and rural area in Scotland. We focus on three questions: (i) what types of personal and social interaction are observed between older adults with chronic pain and their health and social care providers during home visits?; (ii) what aspects of personal and social interaction do rural older adults with chronic pain experience and value?; and (iii) how might eHealth technology have a role to play in future delivery of health and social care services?

## Background

Demographic ageing is occurring worldwide and issues associated with the growing numbers of older people are receiving attention from academics, service providers, and policy makers alike. The percentage of people older than age 65 in the United Kingdom will rise from 17.2% currently to 22.4% by 2032 (Office of National Statistics, 2011). However, the spatial pattern of demographic ageing is not uniform. Urban areas of the Scotland (where approximately 80% of the population live) have the lowest median age whilst remote rural areas are demographically older (Philip, Brown, & Stockdale, 2012); similar patterns are evident in the USA (Philip et al., 2012) and mainland Europe (Dwyer, Baldock, Beaufoy, Bennett, Lowe & Ward. 2002). Demographic ageing in the United Kingdom has, to date, been most pronounced in rural areas and population projections suggest this pattern will continue (Blake, 2009). In Scotland, the proportion of the population aged older than age 75 is predicted to be 26% by 2035 (National Records of Scotland, 2012). However, the proportion of over 75s living in many of the remote rural areas of Scotland will be much higher (National Records of Scotland, 2012). In rural Scotland, provision for older adult care must address several key challenges, including relatively small numbers of service recipients scattered over a wide geographical area and the difficulties of attracting and retaining specialist staff (Cleland, Johnston, Walker, & Needham, 2012; Wilson et al., 2009). An inevitable consequence of demographic ageing is that increasing numbers of older people will make greater demands upon health and social care services.

Many older people living with multiple health conditions suffer from chronic pain, a long-standing symptom estimated to affect 14% of the UK population (Health Improvement Scotland, 2012), although the prevalence of chronic pain has been reported elsewhere to be more than double of this (Azevedo, Costa-Pereira, Mendonça, Dias, & Castro-Lopes, 2012; Reitsma, Tranmer, Buchanan, & Van Den Kerkhof, 2012). Chronic pain has been defined as, continuous, long-term pain of more than 12 weeks or after the time that healing would have been thought to have occurred in pain after trauma or surgery (The British Pain Society, 2013). Pain is one of the most common symptoms of disease and the most frequent complaint reported to doctors. Pain patients are more likely to access health services and the incidence of chronic pain is higher in rural than urban areas (Hoffman, Meirer, & Council, 2002; Tripp, Van Den Verkhof, & McAlister, 2006).

The effects of chronic pain are physical and psychological, impacting upon quality of life and linked to depressive symptoms (Parmelee, Katz, & Lawton, 1991; Power, Perruccio, & Badley, 2005). Clarke and Iphofen (2008) observed that increased social isolation was a concomitant feature of chronic pain. Older people with pain are often worried about becoming addicted to or reliant upon pain-relieving medications, *being a burden*, or being labelled as a *complainer* (Goodman, Hiniker, & Paley, 2003). Increased social isolation and limited opportunities to attend pain support groups (which tend to be located in urban areas) may lead to older rural people being more aware of their pain, especially if they live alone (Pennebaker, 2000).

Health and social policy in the United Kingdom aims to promote active ageing and supports the provision of home-based care to enable independent living for as long as possible (Potter, 2009). eHealth-based care can be used in the home and might help older adults to live independently for as long as possible (in line with the “extitution” model of care favoured today, cf.; Milligan, Roberts, & Mort, 2011). It has considerable potential as a means of supporting independent living among the older population and is preferred by service providers because the cost of eHealth is likely to be less than that of existing modes of delivery. ICT is ubiquitous in everyday life. Although use of personal computers, tablets, and smart phones is lowest amongst the older population, the proportion of regular older users is increasing. Familiarity with ICT in one's personal life is likely to make acceptance of ICT applications in health and social care more probable.

In 2012, the UK Government's Department of Health launched the “3millionlives” initiative (see 3millionlives.co.uk), with the aim of delivering telehealth technologies to 3 million people across England by 2017, potentially saving around £1.2 billion per year. The Scottish Public Health Network (2013) has also explicitly recommended greater use of eHealth solutions in the delivery of care for older people. The focus of eHealth activity is moving rapidly towards the active deployment of this technology. Some, however, have cautioned that the roll-out of eHealth initiatives should not lose sight of scalability challenges particular to rural communities, such as connectivity, skills, and manpower to support IT developments in sparsely populated areas (Roberts, Garrett, & Godden, 2012; The Scottish Government, 2008).

Maintaining social networks and engaging in social activities are important elements of active ageing as older people's social networks contract; the oldest-old may rarely engage in social activities out with the home. Kivett, Stevenson, and Zwane (2000) observed that very old adults have few visits from friends and neighbours. Difficulties in maintaining social connections will be exacerbated for older adults living with chronic pain whose ability to get out and about, entertain visitors, and maintain contact with friends and family can be impaired by their medical condition. In the more remote rural areas these difficulties are further compounded by the dispersed nature of settlements, accessibility challenges, and the fact that friends and family members may not live within easy travelling distance. For many older rural adults living with chronic pain, the only regular in-person social interaction they have is with a health or social care provider. Older adults are concerned that the introduction of eHealth technologies will pose a threat to this relationship (Farmer, Philip, King, Farrington, & MacLeod, 2010).

Recent literature has highlighted the need for health and care technology for older people to be more diverse in design, unique, and circumstance specific (Greenhalgh et al., 2013). Indeed, some have suggested that the use of current eHealth technologies can disrupt face-to-face interaction within the home both with health care professionals and between family members/spouses (Cartwright et al., 2013). For those who do not live alone, new assisted-living systems should therefore be designed to actively promote communication, if more is known about older adults’ current experiences of and preferences for personal and social interaction.

## Method

### Study design

This paper considers the following three questions: (i) What types of personal and social interaction may be observed between older adults with chronic pain and their health and social care providers during home visits? (ii) What aspects of personal and social interaction do rural older adults with chronic pain experience and value? and (iii) How might technology have a role to play in future delivery of health and social care? These questions were explored through our case study research undertaken in remote rural Scotland.

The case study area was classified by the Scottish government as being a “very remote rural area.” It is an island off the west coast of Scotland located in the National Health Service (NHS) Highland region and there was no eHealth activity in the area during the period of data collection. The area was selected purposively following (i) a scoping study of eHealth activity across rural Scotland and (ii) discussions with NHS Highland pain management clinicians, which helped map the prevalence of chronic pain in this large but sparsely populated area (NHS Highland covers 32,500 km^2^ and is home to 320,000 people—www.nhshighland.scot.nhs.uk/AboutUs/Pages/AboutUs.aspx; the case study island has a population of approximately 10,000 people and covers 1656 km^2^). National Records of Scotland 2011 mid-year estimates reported that a fifth of the case study area's population was aged 65+. The 2001 Census reported that 21.3% of the island's population had a limiting long-term illness which is known to correlate with the incidence of chronic pain.

### Provider participants

All GP Practice Managers in the case study area were written to inviting their participation should they have suitable patients. Social carers were then identified once relevant patients were recruited. All health and social care providers received information sheets and signed consent forms prior to the research commencing. Initial interaction about the study commenced by telephone, followed by a visit in person by one member of the research team. Four professional participants were female and one participant was male. The researchers had no control over the attributes of the professional who cared for the older participants in the study.

### Patient participants

Patient participants were recruited through GP Practice Managers, community nursing teams, and the island's Social Care Team. As the intention was to invite both patients and their health/social care provider to participate in the research, the cooperation of these professional groups locally was crucial. The inclusion criteria for the study were that patient participants should:

- be aged between 60 and 79;
- experience chronic pain;
- receive regular (weekly/daily) home visits from health and/or social care staff;
- live in a remote rural location; and
- not use any form of health-related technology to manage their pain.

Eight older adults who met the research inclusion criteria were identified, of whom seven were considered suitable participants in the study by the community nurses/social care team. Only one older adult declined the invitation to take part. In total, there were six patient participants.

By chance, all of the older adults who participated in the study were female. They suffered from a variety of illnesses (including osteoarthritis, Parkinson's disease, arachnoiditis, multiple sclerosis, spondylitis, and severe pain following a road traffic accident) that left them in chronic pain. Three lived alone, three with a spouse. The experiences of chronic pain varied in terms of both the types of pain they suffered from and the length of time they had had their symptoms. The frequency of home visits, interaction with carers, and activities undertaken within the home also varied. The homes of all participants had undergone varying levels of changes to adapt to the individual's daily routine/care challenges. We have therefore been able to capture a wide range of experiences of living with chronic pain in this case study.

All patient participants had limited pain management options available to them, largely because attending either NHS or other formal services or patient support activities would require a very long journey. None had personal experience of their NHS areas’ pain management clinic, which is based over 100 miles away in Inverness. The journey would be too physically demanding and, for those reliant on public or patient transport, logistically challenging to organise.

#### Procedure

Data collection involved detailed observation of a home visit followed by separate semi-structured interviews with the older adult and their health or social care provider. For consistency, interviews and observations were conducted by the same researcher. Six home visits were observed, six older adults were interviewed, and five professionals (one cared for two patients)—three community nurses and two social care providers—were interviewed.

#### Observation

In this research, we wished to capture the nature of both “formal” and “informal” interactions during home visits. Home visits occur for a variety of health and social care reasons. Alongside “formal activities,” a variety of other informal types of social and personal interaction also occur during visits to patient homes. We adopted the following definition of social and personal interaction: the process by which two or more individuals act in response to another's action or behaviour. The response is considered social if the individual takes into account another individual's behaviour which therefore orientates the response (Blumer, 1966). Two structured methods were used to record information about what happens during a home visit—a social and personal interaction observation schedule and the Two-Dimensional Social Interaction Scale (2DSIS).

An observation schedule (see Table I) was devised to record the various types of social and personal interactions that take place during home visits. Observations lasted between 30 and 75 min and interactions were recorded as being either *professional* (interaction directly related to the delivery of health or social care such as discussing symptoms, performing a medical task) or *social* (interaction outside formal care delivery such as asking about friends and family or offering reassurance) and *physical* or *verbal*. Physical activity included clinical touch (e.g. changing dressings, recording blood pressure), social norms (e.g. shaking someone's hand), and reassurance touch (e.g. touching someone's hand to show compassion). Verbal activity included, for example, talking about something clinical, social norms conversation, and friendship level chatting.

**Table I:** Social and personal interaction observation structure

	Type of interaction	Interaction instigated by	Content/comments		Type of interaction	Interaction instigated by	Content/comments
Professional activity	Humour	H&SCP or P		Social activity	Humour	H&SCP or P	
	Affection	H&SCP or P			Affection	H&SCP or P	
	Dislike	H&SCP or P			Dislike	H&SCP or P	
	Clinical touch	H&SCP or P			Clinical touch	H&SCP or P	
	Social norms touch	H&SCP or P			Social norms touch	H&SCP or P	
	Reassurance touch	H&SCP or P			Reassurance touch	H&SCP or P	
	Verbal	H&SCP or P			Verbal	H&SCP or P	
	Listening	H&SCP or P			Listening	H&SCP or P	

H&SCP=health and social care professional, P=patient.

A pre-tested 2DSIS (Tse & Bond, 2001) was used to provide an overall perspective of each observation. The purpose was not to quantify interaction but to identify the types and level of interactions and communication taking place between the older adult and their health or social carer within the home. Table II provides details of the 2DSIS.

**Table II:** Two-dimensional social interaction scale: types of social interaction

Health and social care provider		Patient	
Active participation Friendly Spontaneous Talkative Energetic	Passive participation Agreeable Considerate Attentive Co-operative	Active participation Friendly Spontaneous Talkative Energetic	Passive participation Agreeable Considerate Attentive Co-operative
Active non-participation Self-centred Insensitive Self-interested Arrogant Irritating	Passive non-participation Indifferent Aloof Detached Quiet Demanding Reserved Undemanding	Active non-participation Self-centred Insensitive Self-interested Arrogant Irritating	Passive non-participation Indifferent Aloof Detached Quiet Demanding Reserved Undemanding

After Tse and Bond, 2001.

#### Interviews

Older adults and their health or social care professionals were interviewed separately using a semi-structured interview schedule with relevant prompts. Interviews with patients immediately followed the home visit observation; interviews with health professionals were conducted at a mutually convenient time thereafter. Patient interviews lasted between 30 and 55 min, with health professional interviews lasting between 20 and 60 min. The interviews provided opportunities for opinions about the non-clinical benefits of in-person care delivery to be elicited. Participants were also invited to reflect on their experiences of and attitudes towards the use of technology in their private lives and in their “medicalised” or professional lives as well as how they thought technology could play a role in the future delivery of health and social care services, in general, and for older people living with chronic pain, in particular.

All the interviews were digitally recorded and transcribed verbatim. Analysis adopted an iterative framework approach (Yin, 2003) involving familiarisation with the data, identification of a thematic framework, indexing, charting, and finally, mapping and interpreting the findings. Transcripts were coded independently by three members of the TOPS research team, and the coding framework was then developed collaboratively. Field notes were also recorded after each interview and observation. All field notes and interview transcripts were managed and analysed in QSR NVivo 9.

The information collected during home visit observations and the interviews with older participants and with their health or social carers addressed the three research questions. Firstly, the home visit observation schedule and associated field notes provide the basis for reflections on the types of personal and social interaction that may be observed during a home visit. Secondly, the interviews identified the types of personal and social interaction valued by older adults with chronic pain. Thirdly, interview responses from participants and health and social care professionals are drawn upon to consider how technology could have a role to play in the future delivery of health and social care services.

## Results

### What types of personal and social interaction may be observed between older adults with chronic pain and their health and social care providers during home visits?

Home visit observations revealed the range of activity and social interaction taking place during a home visit and information about the level of support the health or social carer provides to the patient. Many of the interactions observed between the health professionals and patients were clinically orientated, involving clinical touch such as taking blood pressure readings or changing wound dressings. Social care duties mainly involved personal care such as moving and handling (helping the patient get up or be put into bed; making them comfortable in a chair and ensuring that things they needed were close at hand). Other household activities were also observed during a home visit such as the health/social carer washing up crockery or bringing in the post; though these activities were mainly undertaken by a spouse or visiting family member. Although such activities were not specifically falling within their job description, such acts of “good will” can be extremely helpful for those spouses going through well-documented transition and identity changes from the role of husband/wife to the role of spousal carer (Aneshensel, Pearlin, & Schuler, 1993).

It was evident that home visits had a positive impact on the patient's opportunity to maintain social and personal interaction. Light-hearted discussions were observed, often using humour, and conversations included talking about what was happening in the community, exchanging news about family and friends and reminiscing about the past, sometimes about when patients were younger or had more active involvement in the community. Allowing an older adult to remain engaged with what is going on in their community helps them to retain a sense of belonging even if they are rarely away from their home.

All health and social care professionals were observed engaging in active participation activities during home visits: all were friendly and they were mostly talkative. No active non-participation was observed (i.e. self-centred, insensitive, self-interested, arrogant, or irritating behaviours). Passive participation was observed occasionally, when both (health and social) professionals were attentive to what the older adult said or did. There was one instance of passive non-participation, where a professional was considered to be detached throughout the entire home visit.

Older adults actively participated in the home visit. Four were observed to be both friendly and talkative and none were actively non-participatory. More signs of passive participation and non-participation were observed than active participation. For example, passive non-participation interactions were observed during five visits, where participants were detached, quiet, reserved, and indifferent. Passive participation was observed in three visits where participants were agreeable and cooperative. Our findings suggest that care professionals are overtly friendly and verbally engaged with the older people they visit at home. Most of the older adults responded to this by being actively friendly and communicative in return. The degree of pain being experienced by participants undoubtedly influenced levels of passive non-participation, with professionals mentioning during interviews that the nature of the interaction during home visits varied according to how a patient was feeling. In cases where chronic pain was evidently severe, the use of humour was often used to try and lighten the situation or take the patient's mind off their pain. Humour was observed being instigated by both the patient and the health/social carer. Reassurance touch was also observed in such instances.

### What aspects of personal and social interaction do rural older adults with chronic pain value?

Living with chronic pain can make it difficult to maintain social connections and be able to contribute to, and participate in, the local community. Some of the difficulties are universal to all chronic pain patients, others are particular to or made more challenging in a rural context. The changing nature of relationships with friends and family and the various ways in which pain inhibits the ability of the older adult to engage in different modes of social interactions were the most common themes to emerge from the analysis.

Patients discussed the changing nature of social interactions with those who visit the family home and described how they felt friends are often embarrassed or do not know what to say when they visit. When asked about whether friends still come and visit, one participant who had severe chronic pain and mobility challenges replied:

Most of them. Most of them – I think its embarrassment, I don't think they know what to say to me or how to act around me. (Participant 4)

Reasons for visiting often change. The social element that was once central to the relationship between friends can be replaced by “acts of friendship.” For example, we were told of friends who visited to help out in the home, drop off cooking or help with chores.

Well I have very good friends who do my shopping. Another one does my housework, another one will cook or bring some meals that she's cooked at home for me to put in the freezer and I'm really lucky with friends. I have visits every single day … I've always plenty friends. (Participant 6)

During periods of intense chronic pain, patients described feeling reluctant to talk or communicate with others, including their health or social care providers. One social carer explained that she knew not to interact with her patient when she was in intense pain, as the patient preferred silence. Such a lack of engagement was not a reflection of the older adult's lack of sociability in general, but an inability to engage with others when pain is intense can put a strain on personal relationships and make friends and family reluctant to visit or attempt to interact with the older adult. Some patients told us that they have to limit visits from friends because visits from or other forms of social contact with friends could be tiring.

I like finding out things and I like other people but I don't like – I used to like people and I used to have a lot of fun talking with people but it's got now that if I'm talking a long time then the night and the next day and – I'm in a worse state. (Participant 3)

Health professionals were also aware of the strain maintaining social interactions can create.

She sat at Christmas last year and wrote 60 Christmas cards and ended up in Hospital with emergency admission because she just exhausts herself. So we really had to say to visitors in the community just to back up her husband to say, if you are visiting it's a limited visit – time yourself. Don't be just sitting chatting on and on and on because she can't. (Health Professional 1)

If she interacts with folk for too long then that will tire her out and exhausts her and makes things more of a problem. Again, she has to adapt to that and that probably bothers her perhaps more than she says. (Health Professional 2)

We were also told about the difficulties chronic pain patients can face in trying to retain engagement with community life and participating in activities that take place outside the home. For example, one participant described not being able to go to church regularly because of the tiring journey and the fact that the church pews were very uncomfortable for her to sit on. It saddened her that she could no longer attend church in the way she had been used to doing, feeling that she missed both religious observance and the opportunities for social interaction that going to church offered.

For some older adults with chronic pain, opportunities to socialise with visitors or to leave the home and interact with others were very limited. For these individuals, the home visit provided personal contact that otherwise would be missing from their lives. However, the home visit and the social interaction it brought were also important for those older adults who did not live alone and who had visits from friends and family. The changing nature of their relationships with others, directly related to their condition, resulted in them feeling that their opportunities for social interaction had declined. The opportunities to “socialise” with their health/social care professional during a home visit helped to “fill a gap.” These findings thus demonstrate the perceived importance of the social dimensions of home visits to older chronic pain patients regardless of personal living arrangements (living alone or with a spouse) or whether or not other people regularly visit the home.

The nature of the relationship between patients and professionals in remote rural areas, where it is likely that the patient and professional are known to each other outwith the care relationship, may have influenced the social interactions we observed during home visits. Fewer day-to-day opportunities for face-to-face interaction with other people in sparsely populated areas may also make the social interactions that take place during a home visit even more important for rural older people. The importance of pre-existing familiarity between older adults and health/social care professionals to home-visit-based social interactions and the associated promotion of broader well-being would be worth further exploration. It has been reported elsewhere that health and social care providers do more during home visits for rural patients, sometimes because they know other support services are not available locally or they know that their patient does not have close family or friends nearby who could help out informally. In other words, rural professionals work outwith their job description. This additional support may be the difference between an older person remaining at home or having to move to some form of supported accommodation, a move which in a rural context often entails leaving one's “home” community (Farmer, West, Whyte, & Maclean, 2005). An opportunity to observe “formal” and “informal” activities undertaken by professionals during a home visit is thus useful to understand the nuances of these relationships more thoroughly.

### How might technology have a role to play in future delivery of health and social care services?

Health and social care professionals all offered positive opinions about the current and future use of eHealth, but their first-hand professional experience of using eHealth was limited. Health Professional 1, a community nurse, made favourable comments about online training courses (e-learning) and noted that an e-learning package about elderly care and chronic pain would be useful, particularly for professionals who live and work in remote communities, for whom attending training courses in person is time-consuming because of the distances that must be travelled to reach a training centre. Health Professional 1 was also open to patients using Internet resources to become better informed about their conditions, viewing this as an empowering activity:

… [eHealth]would make such a huge difference … well, the thing is, even for their well-being, it has a knock on effect onto everything else …. (Health Professional 3, social care professional)

We are all into enablement just now, where you get the patient to do as much as possible themselves – the easier it is for the patient to use, the better. (Health Professional 2, community nurse)

Despite many positive attitudes towards eHealth being reported, IT infrastructure challenges restricting the deployment of telehealth in the case study area were mentioned. In the United Kingdom, the “digital divide” means that few remote rural areas have access to fast, reliable broadband (Philip, Cottrill, & Farrington, in press; Royal Society of Edinburgh, 2010, 2013). The use of new eHealth technologies across rural areas is impossible if minimum download and upload speeds are not supported by an area's broadband infrastructure. Both Health Professionals 1 and 2 knew of home-based telehealth having been trialled in their area and discussed connectivity problems that created difficulties in using the technology:

Mr McDonald (a pseudonym has been used) uses tele-health. We have real difficulties getting it to work at Mr McDonald's. Mr McDonald is in a really isolated part of the island and I think there were problems with the phones and there was problems getting it to work effectively …. (Health Professional 1, community nurse)

… but unfortunately it can't connect to the phone lines[telephone lines that support broadband], although it can take the data, it can't transmit it back. (Health Professional 2, community nurse)

Despite participants being broadly positive about and receptive to eHealth, words of caution about two eHealth-related issues were voiced. Health Professional 4, a community nurse, noted that for eHealth-related technology to be beneficial for people with chronic pain the user needs to be engaged, keen to learn, a computer literate, willing, and able to take responsibility for aspects of their care and, importantly, to have good family back up, but not all patients would meet these criteria. Health Professional 4 discussed how the Internet was becoming ubiquitous and how modern technology has changed people's lives, but they were not sure if the positive aspects of eHealth yet outweigh the negatives. Particular concern was expressed about the consequence of replacing face-to-face interaction with technology:

… it depends on how much value that person places on face-to-face interaction. As I said, if they've got very good social back-up and family back-up and they are seeing people on a regular basis then, fine. Or if they are the kind of person who doesn't want to see anybody at all, quite happy on their own – then fine. But if it's somebody who is sitting on their own and have no family or people popping in on a regular basis then I don't think it's going to be of benefit …. (Health Professional 4, community nurse)

Various types of technology were used in everyday life by the older adults with chronic pain we interviewed. Everyone used the telephone and a small number used online Voice over Internet Protocol services (e.g. Skype) to keep in contact with friends and family. The type of ICT used could also influence patient's use of eHealth.

The iPad is much faster, easier and I just sit on my bed with my knees up, not holding it, like this – ‘cos I couldn't, I just rest it. Yeah. Whereas the other [laptop] you seem to have to concentrate, move your mouse – because I can't do it with rolling the finger, I don't like that—the laptop. So I use the mouse of course but it's much, much easier on the iPad. (Participant 6)

One participant had impaired hearing and discussed the challenges she faced using the telephone. Her impairment made it difficult to keep in touch with friends and family who did not live nearby, making in-person interaction all the more important:

Because my hearing aids, they were better and my hearing wasn't so bad and I could keep in touch. And I learnt to lip-read a lot. Sometimes I came near it but sometimes I was that far out we'd just have a good giggle over what I thought we'd said! (Participant 4)

Using various types of ICT can also be physically and mentally tiring. Participants told us that they felt the difficulties they faced using technology has a knock-on effect on their efforts to stay socially connected. They discussed the physical difficulties using the telephone, that active engagement with others on the phone is tiring, and that the concentration levels required can limit the length of time a computer is used for.

I can't hold the receiver for long because of my fingers; I've no strength in my hands. So everybody, again, all my friends know that when I've spoken for 1 or 2 min I've got to put it down and in the middle of something I have to say, “I have to put the phone down,” and nobody bothers because they know what I'm like. (Participant 6)

It exhausts me. Not as bad just now but it's still … you are limited to how long you can talk. How long you can listen, that's part of it. Most of the friends they phone on a regular basis or you phone and they now know that they can have a limited period, they can't go on the way they used to. (Participant 1)

Probably just being able to use a lap top and I've – I can still spell perfectly and that sort of thing. When I say I can't read, it's just concentration. (Participant 1)

Chronic pain patients are encouraged to participate in self-management activities, some of which involve self-help group meetings that can be delivered virtually. We were told about the challenges a participant thought she would face if she were to use online videoconferencing to participate in a self-help group:

I find with my hearing it's very difficult, I couldn't do a group, it would have to be one or two at the most because you are trying to catch up with who said and what did they say and then you are asked and you've got to embarrass yourself by saying, I'm sorry, I didn't catch what was going on. (Participant 4)

Notwithstanding the potential difficulties of participating in pain management clinic activities remotely, an opportunity to attend clinic activities by videoconference could be beneficial for older adults with chronic pain. Technology could also be used to provide remote access on a regular or occasional basis to services such as physiotherapy or cognitive behavioural therapy that are not readily available locally. Physiotherapy from home was not available for the patients we interviewed, despite it being identified by health professionals as something they would benefit from.

## Conclusions

The value and importance of social interaction as an integral part of a home visit has been highlighted. Observations demonstrated that, as a matter of routine, both “professional” *and* social activities take place during home visits. The social interaction taking place as part of a home visit is a powerful tool for maintaining feelings of social connection outside the home and within the wider community. Home visiting relationships between patients and professionals were positive; patients were observed to be determined to interact, often in the face of extreme pain. Older patients valued the sociability of the home visit regardless of whether they also received visits from other friends and family or did not live alone. The home visit was also valued by spouses, carers whose social interaction opportunities can also contract and whose needs can easily be overlooked. Home visits undoubtedly contribute to broader well-being of patients and spouse carers.

For some of the patients we observed the physical presence of a health professional was essential: for example, patients who required clinical activities that cannot be carried out remotely using an eHealth application. Others could, potentially, have some of their needs met through eHealth applications and were positive about the potential use of eHealth technologies to manage chronic pain. However, in light of comments we received, we propose that decisions regarding whether an older person should use eHealth as part of his or her care package should also involve the health/social care professionals who know the individual and can input into service provision decision making regarding who receives care delivered by different delivery modes.

The ICT infrastructure limitations, ergonomic challenges, and difficulties using technology associated with impairments such as hearing loss we identified in our analysis are issues relevant to the development and deployment of eHealth to support older chronic pain patients in the future. Older people are often overlooked in the design of new ICT devices and applications (Rice, Newell, & Morgan, 2007), but ergonomic and other usability challenges (including weight, touch, sight, hearing, the need to type or use a mouse or touch pad, acceptability of interface, etc.) identified in the patient interviews are a reminder of the need to involve older adults, including those with chronic pain, in the design and development of eHealth technologies. Those whose conditions are less severe than the participants in this study might potentially benefit more from eHealth opportunities within their care package. Connectivity, reliability of technology (especially within remote and rural areas), and the cost of purchasing devices required to use eHealth applications will all play a part in future uptake amongst the older population.

While the delivery of professional services can be costed, the value placed on face-to-face care, and the non-professional activities that take place during a home visit, is difficult, if not impossible to quantify yet our study has shown that it is of demonstrable value to patients, especially if the patient lives in sparsely populated remote areas. A balance where digital interaction could enhance rather than replace face-to-face care may be most appropriate. However, this balance must also consider cost-effectiveness and patient well-being.

The TOPS project engaged with patients and their home care providers together. The robust qualitative evidence from this study demonstrates the ways in which older rural people with chronic pain value their social and personal interaction. A limitation of the study is that all patient participants were female. Further research with male participants would assist in indicating whether the experiences of older adults with chronic pain in remote and rural areas differ by gender. A second limitation is that our participants lived in an area where no formal eHealth initiative was running. Opinions about eHealth technology use amongst the older generation may be different in remote and rural areas where eHealth is provided and Internet connectivity is better. Further research in a remote rural area with good ICT infrastructure supporting the use of eHealth applications may uncover different experiences of, and attitudes towards, the use of eHealth technologies.

Our research demonstrates that there is variability in how ready the current generation of older people in remote and rural locations are to deal with opportunities for digital care to enhance face-to-face interaction in terms of acceptability, a physical ability to use technology, and having access to the IT infrastructure necessary to use digital care options. Readiness, at present, should be assessed at an individual level and will inevitably change as the older population become even more technologically able and connectivity issues improve. Overall, our findings show that the potential recipients of eHealth are open to the use of such technologies, that eHealth may provide opportunities to sustain and enhance these interactions but that in-person care is likely to remain an important element of caring for older people with chronic pain in the future.
